# The prevalence of Brugada syndrome in patients presenting to an emergency room in Riyadh, Saudi Arabia

**DOI:** 10.3389/fcvm.2026.1871087

**Published:** 2026-09-15

**Authors:** Mudafr Alkhedr, Layan Alabbas, Sarah Alfarraj, Faisal Alshehri, Rayan Jabaan, Meshal Alhamed, Mohammed Alfaris, Mohammad Alkhorijah, Sulaiman Alshowihi, Fahad Abuguyan, Wael Alqarawi

**Affiliations:** 1 Department of Internal Medicine, King Fahad Medical City, Riyadh, Saudi Arabia; 2 College of Medicine, King Saud University, Riyadh, Saudi Arabia; 3 Prince Sultan Military Medical City, Riyadh, Saudi Arabia; 4 Emergency Department, King Saud University Medical City, Riyadh, Saudi Arabia; 5 Department of Cardiac Sciences, College of Medicine, King Saud University, Riyadh, Saudi Arabia; 6 King Fahad Cardiac Center, King Saud University Medical City, Riyadh, Saudi Arabia; 7 National Center for Sudden Death, King Saud University Medical City, Riyadh, Saudi Arabia

**Keywords:** Brugada pattern, Brugada syndrome, emergency department, prevalence, Saudi Arabia

## Abstract

**Background:**

Brugada syndrome (BrS) is a rare inherited arrhythmia associated with an increased risk of sudden cardiac death. Significant variation in the prevalence of BrS across different countries has been well documented. However, no study has examined the prevalence of BrS in Saudi Arabia. Therefore, our aim was to assess the prevalence of BrS in patients presenting to an emergency department (ED) in Riyadh, Saudi Arabia.

**Methods:**

This retrospective study included consecutive adult patients who presented to the ED at King Khalid University Hospital (KKUH) and underwent an electrocardiogram (ECG). Demographic and clinical variables were collected from the records, and ECGs were examined for the presence of a Brugada pattern (BrP). BrS was diagnosed when a type 1 BrP was observed with no secondary causes, and BrP was diagnosed when a non-type 1 pattern was observed. A prespecified sensitivity analysis was conducted, excluding patients whose presentation was potentially compatible with BrS (syncope, presyncope, cardiac arrest, and palpitations).

**Results:**

We included 1,000 patients. Men constituted 498 patients (49.8%). The majority were Saudi nationals (830, 85.6%) and the overall median age was 46 years (IQR: 30–65 years). The majority of the patients were afebrile at presentation (915, 93%) and a small number of patients took Brugada-inducing medications (27, 2.7%). The prevalence of BrS was 2/1,000 (0.002; 95% CI: 0.0002–0.007) and the prevalence of BrP was 26/1,000 (0.026; 95% CI: 0.0179–0.0390). Among the patients with BrP, 11 exhibited a type 2 pattern (42.3%) and 15 exhibited a type 3 pattern (57.7%). The sensitivity analysis revealed a prevalence of BrS of 2/912 (0.002; 95% CI: 0.0–0.0061) and a prevalence of BrP of 26/912 (0.028; 95% CI: 0.0178–0.0402).

**Conclusion:**

This is the first Saudi Arabian study on BrS prevalence, suggesting a high prevalence and a notable proportion of non-type 1 Brugada electrocardiogram (ECG) patterns.

## GRAPHICAL ABSTRACT

**Figure:**
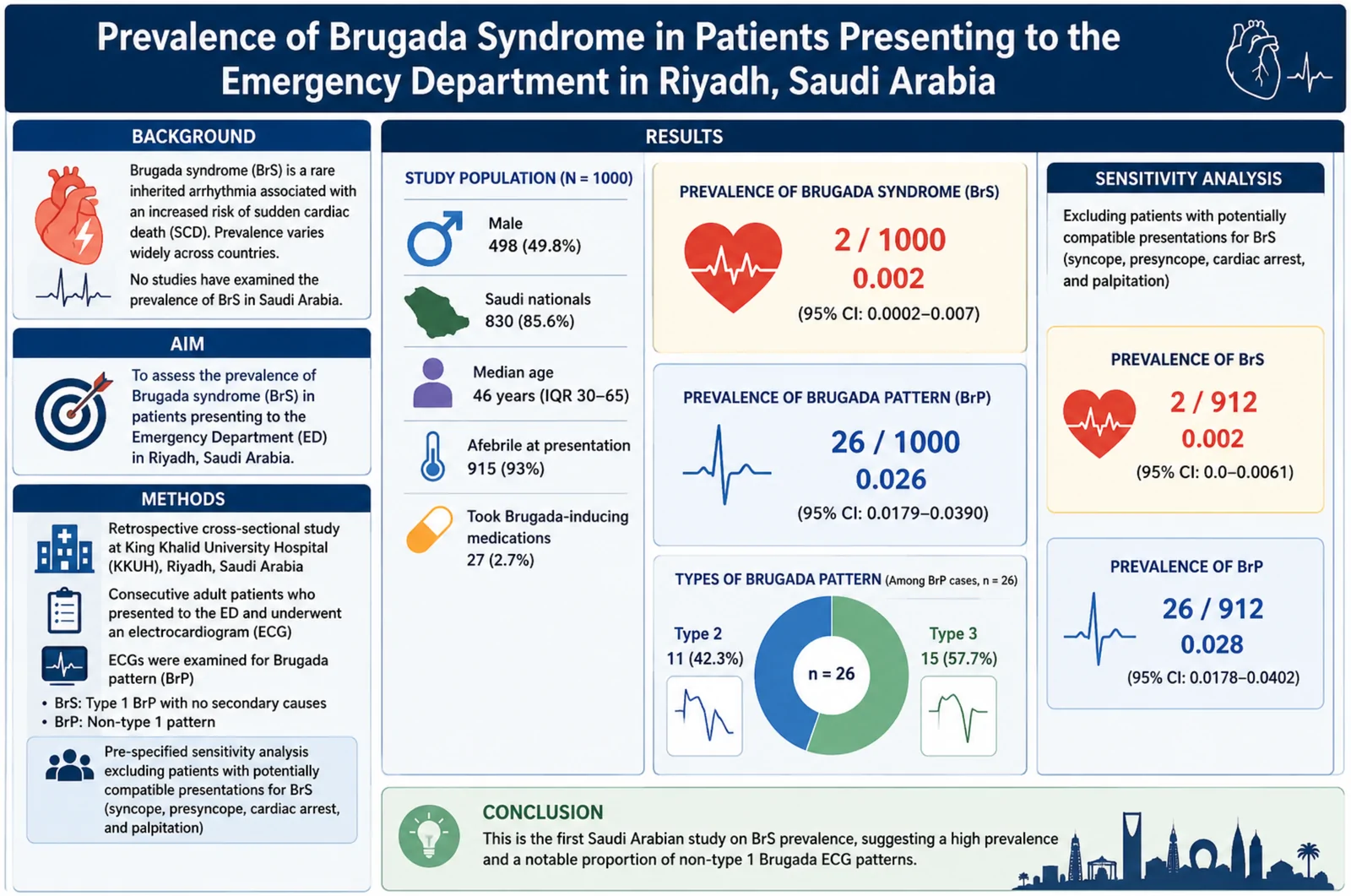

## Introduction

Brugada syndrome (BrS) is an inherited cardiac disorder characterized by distinct electrocardiographic patterns and an increased risk of ventricular arrhythmias, including sudden cardiac death (SCD). The description of the clinical presentation of what is now known as Brugada syndrome first appeared in a medical publication by Professor Maria Paz Mendoza-Guazon in 1917, describing young males who were in apparent health and died suddenly during sleep (1, 2). Later, in 1953, the ECG pattern was described as a mimic of myocardial infarction (3). The Brugada brothers then, in 1992, described the specific ECG pattern associated with SCD as a syndrome (4). Since then, significant progress in the understanding of the pathophysiology of this condition has been made. It is now believed that BrS involves impaired sodium channel function. A loss-of-function mutation in *SCN5A*, which encodes the Nav1.5 sodium channel, is the only genetic defect considered to be disease-causing and is responsible for approximately 20% of cases (5). Other implicated genes involving sodium, potassium, and calcium channels have been disputed (6). However, common genetic variants leading to decreased expression of sodium channel function have been described and explain familial segregation in those without *SCN5A* variants (7, 8).

Understanding the prevalence of BrS is crucial for proper clinical decision-making, public health policy development, and healthcare planning. Assessing the prevalence is particularly important for BrS given its well-documented variance in prevalence among different ethnicities and countries (9, 10). Indeed, a systematic review of the worldwide prevalence of BrS reported a wide prevalence range, with an overall prevalence of 0.5 per 1,000 individuals (0.0005), ranging from 1.8 per 1,000 individuals (0.0018) in Southeast Asia to 0 per 1,000 individuals in North Africa. Moreover, BrS in Asians was nine times more common than in those of white ethnicity and 36 times more common than in Hispanics (10). There is a paucity of data pertaining to those of Arab ethnicity and Saudi Arabia. Therefore, the aim of this study is to assess the prevalence of BrS in Saudi Arabia.

## Methodology

### Study population

Our study used a retrospective cross-sectional design. The target population consisted of consecutive adult patients [age > 14 years, per institutional policy at King Khalid University Hospital (KKUH)] who presented to the emergency department (ED) at KKUH and underwent an electrocardiogram (ECG), starting in December 2023 and proceeding retrospectively. The prespecified sample size of 1,000 was based on the international prevalence of BrS (10).

Only patients with missing ECGs or file notes were excluded. Demographics and clinical variables were collected from the records, and ECGs were examined for the presence of a Brugada pattern, which was verified by an expert (WA).

To minimize selection bias, a prespecified sensitivity analysis was performed, excluding patients with symptoms compatible with BrS (syncope, presyncope, palpitations, and cardiac arrest).

### Study variables

We collected relevant data, including patients' demographics. clinical and medical history {febrile state, drugs including those known to induce BrP, and past medical history [e.g., hypertension (HTN), diabetes mellitus (DM), dyslipidemia (DLD), stroke, and atrial fibrillation (A.fib)]}, presenting symptoms (e.g., palpitations, presyncope, syncope, cardiac arrest, or road traffic accident injuries), ECG parameters (Brugada pattern/type), final diagnosis, and laboratory measurements (electrolyte panel at the time of ECG recording).

### Criteria of diagnosis

BrS is diagnosed when a typical type 1 Brugada ECG pattern is detected in any of the right precordial leads (V1–V3) in the absence of a condition that may explain this pattern (phenocopy). A typical type 1 BrP is characterized by coved-type ST-segment elevation ≥2 mm followed by a negative T wave (4, 11–17).

A Brugada ECG pattern is defined when there is >2 mm J-point elevation followed by >1 mm saddleback-shaped ST-segment elevation (type 2) or <1 mm of any shape (type 3) (3–8).

### Statistical analysis

Continuous data are presented as median (interquartile range) and categorical data as numbers (percentages). The prevalence of BrS and BrP is presented with 95% confidence intervals. Comparisons were done using the Wilcoxon rank-sum test for continuous variables and the chi-square test or Fisher's exact test for categorical variables. The statistical analysis was performed using SAS (SAS Institute, NC, USA).

### Ethical considerations

This study was conducted in accordance with the Declaration of Helsinki and approved by the Institutional Review Board of the College of Medicine, King Saud University, Riyadh, Saudi Arabia (IRB approval number E-23-8042).

## Results

### Overall population

We included 1,000 patients. Among them, 28 patients had a Brugada ECG pattern and 972 did not. Men constituted 498 patients (49.8%). The majority were Saudi nationals (830, 85.6%), and the overall median age was 46 years (IQR: 30–65 years). Prevalent comorbidities included HTN (357, 35.7%), DM (338, 33.8%), DLD (143, 14.3%), CAD (143, 14.3%), stroke (84, 8.4%), and A.fib (69, 6.9%); family history of sudden cardiac death (FHx of SCD) was rare (2, 0.2%). The majority of the patients were afebrile at presentation (915, 93%). A small number of patients took Brugada-inducing medications (27, 2.7%), namely amitriptyline, flecainide, bupivacaine, and propofol. Further baseline characteristics are presented in Table 1.

**Table 1:** Baseline characteristics of patients with and without a BrP.

Variable	Brugada pattern (*N* = 28)	No Brugada pattern (*N* = 972)	All patients (*N* = 1,000)	*P*-value
Sex (male), *n* (%)	16 (57.1)	482 (49.5)	498 (49.8)	0.430
Nationality (Saudi), *n* (%)	24 (96)	806 (85.3)	830 (85.6)	0.134
Age, median (IQR)	47 (31–67)	46 (30–65)	46 (30–65)	
Brugada syndrome, *n* (%)	2 (7.1)		2 (0.2)	
Brugada pattern (Type 2), *n* (%)	11 (39.2)		11 (1.1)	
Brugada pattern (Type 3), *n* (%)	15 (53.5)		15 (1.5)	
Febrile state (afebrile), *n* (%)	26 (92.8)	889 (93.1)	915 (93)	0.945
Hypertension, *n* (%)	12 (42.8)	345 (35.6)	357 (35.7)	0.432
Diabetes mellitus, *n* (%)	14 (50)	324 (33.4)	338 (33.8)	0.068
Dyslipidemia, *n* (%)	8 (28.5)	135 (13.9)	143 (14.3)	0.029
Stroke, *n* (%)	2 (7.1)	82 (8.4)	84 (8.4)	0.801
Atrial fibrillation, *n* (%)	4 (14.2)	65 (6.7)	69 (6.9)	0.120
Coronary artery disease, *n* (%)	9 (32.1)	134 (13.8)	143 (14.3)	0.006
FHx of sudden cardiac death, *n* (%)	0	2 (0.2)	2 (0.2)	0.949
Sodium level (normal), *n* (%)	13 (61.9)	703 (86.6)	716 (86)	0.0009
Chloride level (normal), *n* (%)	12 (57.1)	697 (86.2)	709 (85.4)	<0.001
Potassium level (normal), *n* (%)	18 (94.7)	734 (91.7)	752 (91.8)	0.624
Calcium level (normal), *n* (%)	10 (76.9)	404 (84.8)	414 (84.7)	0.493
Toxicology screen (positive), *n* (%)	0	3 (4.4)	3 (4.4)	0.793
Taking medications that induce a Brugada pattern, *n* (%)	0	27 (2.8)	27 (2.7)	0.382

### Prevalence of BrS

Two patients had BrS. Table 2 describes these BrS patients. Patient 1 was a 45-year-old afebrile Saudi man who presented with shortness of breath and a productive cough. Patient 2 was a 36-year-old afebrile non-Saudi woman who presented with chest discomfort. Neither patient had comorbidities (including HTN, DM, DLD, stroke, A.fib, or CAD) nor any known exposure to BrP-inducing medications. Both patients were discharged from the ED with a referral to electrophysiology clinics for further evaluation. Patient 1 attended follow-up and was subsequently diagnosed with BrS, whereas Patient 2 did not attend follow-up due to eligibility issues (non-national).

**Table 2:** Baseline characteristics of the patients with BrS.

Variable	BrS patient 1	BrS patient 2
Sex	Male	Female
Nationality	Saudi	Non-Saudi
Age	45	36
Presenting symptoms	Shortness of breath with productive cough	Chest discomfort
Febrile state	Afebrile	Afebrile
Hypertension	No	No
Diabetes mellitus	No	No
Dyslipidemia	No	No
Stroke	No	No
Atrial fibrillation	No	No
Coronary artery disease	No	No
Sodium level (mmol/L)	133	142
Chloride level (mmol/L)	95	104
Potassium level (mmol/L)	3.74	4
Calcium level (mmol/L)	2.35	2.29
Taking medications that induce a Brugada pattern	No	No

### Prevalence of BrP

Among the 26 patients with BrP, 11 exhibited a type 2 pattern (42.3%) and 15 exhibited a type 3 pattern (57.7%). This group comprised 15 men (57.7%), with a median age of 47 years (IQR: 31–67). In total, 23 (88.5%) of these patients were Saudi and the majority were afebrile (24, 92.3%). No patient had a FHx of SCD or took medications known to induce BrP. Further baseline characteristics are presented in Table 3.

**Table 3:** Baseline characteristics of the patients with a Brugada ECG pattern.

Variable	Patients with a Brugada ECG pattern (*N* = 26)
Sex (Male), *n* (%)	15 (57.7)
Nationality (Saudi), *n* (%)	23 (88.5)
Age, median (IQR)	47 (31–67)
Brugada pattern (Type 2), *n* (%)	11 (42.3)
Brugada pattern (Type 3), *n* (%)	15 (57.7)
Hypertension, *n* (%)	12 (46.2)
Diabetes mellitus, *n* (%)	14 (53.8)
Coronary artery disease, *n* (%)	9 (34.6)
Dyslipidemia, *n* (%)	8 (30.8)
Atrial fibrillation, *n* (%)	4 (15.4)
Stroke, *n* (%)	2 (7.7)
Febrile state (afebrile), *n* (%)	24 (92.3)
Sodium level (normal), *n* (%)	12 (60)
Chloride level (normal), *n* (%)	11 (55)
Potassium level (normal), *n* (%)	16 (94.4)
Calcium level (normal), *n* (%)	8 (72.7)
FHx of SCD, *n* (%)	0 (0)
Taking medications that induce a Brugada pattern, *n* (%)	0 (0)

### Sensitivity analyses

We performed a sensitivity analysis to minimize hospital-setting bias. This was done in an attempt to make the patients enrolled in this study more representative of systematic screening in the general population (i.e., BrS is diagnosed incidentally rather than being the cause of presentation to the ED), which revealed a BrS prevalence of 2/912 (0.002; 95% CI: 0.0–0.0061) and a BrP prevalence of 26/912 (0.028; 95% CI: 0.0178–0.0402).

## Discussion

This study represents the first investigation into the prevalence of BrS and BrP in Saudi Arabia, revealing a BrS prevalence of 0.002 (95% CI: 0.0002–0.007) and a BrP prevalence of 0.026 (95% CI: 0.0179–0.0390) among patients presenting to an ED. It is important to note that our study population consisted of patients who presented to the emergency department and underwent ECG evaluation, which differs from the general population. These findings offer some insights into the regional epidemiology of BrS in the Middle East, a region where comprehensive prevalence data have historically been limited. Our observed BrS prevalence exceeds the pooled global prevalence of 0.0005 (0.5/1,000) reported in Vutthikraivit et al.'s 2018 meta-analysis of 61 studies (10), and is higher than that reported in Western populations (typically 0.0001–0.0003 in European/North American cohorts) (10, 13). However, it remains lower than that reported in high-risk Southeast Asian populations, where BrS prevalence reaches 0.0037 (10), consistent with established ethnic gradients wherein BrS is ninefold more common in Asians than those of white ethnicity (10). Similarly, the observed type 2/3 BrP prevalence of 0.026 is 4.3-fold higher than the global average of 0.0061 (10), but below Southeast Asia's reported prevalence of 0.0355 (10). This epidemiological pattern may reflect Saudi Arabia's unique genetic admixture, bridging white ethnicity and Asian ancestry; notwithstanding the small numbers, this warrants investigation into population-specific variants in *SCN5A* and other susceptibility genes implicated in BrS pathogenesis (14).

To contextualize our results further, it is important to examine existing studies from neighboring Middle Eastern countries (Table 4). A study by Babaee Bigi et al. in Iran evaluated patients presenting with palpitations in southern Iran and revealed similar findings. A Brugada-type ECG pattern was reported in 0.0256 of 3,895 patients (18). Of these patients, 14 (0.0035) demonstrated a spontaneous type 1 pattern. In contrast, an Egyptian study reported a low prevalence of Brugada-type ECG patterns (0.0055) in a consecutive hospital-based cohort of 4,000 subjects, with all cases being the saddleback type (type 2/3) and no type 1 patterns were observed (19). Unlike our study, this study included pediatric patients, which may explain the lower prevalence since BrP typically manifests in adulthood (22, 23).

**Table 4:** Comparison with previous Middle Eastern studies.

Study	Country	Population	Sample size	Mean age	Prevalence of BrS, *n* (%)	Prevalence of type 2/3 BrP, *n* (%)
Babaee Bigi et al. (18)	Iran	Patients presenting to ED with palpitations	3,895	38.2 ± 11.9 years	14 (0.36)	86 (2.2)
Akram et al. (19)	Egypt	Patients admitted with a non-cardiac presentation	4,000	Nil	0	22 (0.55)
Bozkurt et al. (20)	Turkey	General population	1,238	38.9 ± 17.6 years	1 (0.08)	5 (0.4)
Adler et al. (21)	Israel	Febrile vs. afebrile patients presenting to the ED	402 vs. 909	62 ± 22 years vs. 61 ± 19 years	8 (2) vs. 1 (0.1)	7 (1.7) vs. 4 (0.4)

Moreover, a study by Bozkurt et al., conducted in Turkey, reported a Brugada-type ECG pattern frequency in one patient (0.0008), who demonstrated a type 1 pattern (20). This is lower than our findings and may indicate a different genetic architecture of the Turkish population compared with ours.

In addition, our findings are consistent with previously published studies examining the prevalence of the type 1 Brugada ECG pattern. Notably, Adler et al. reported a baseline prevalence of approximately 0.1%, which is comparable to the prevalence observed in our cohort and in other similar studies (21).

Beyond these regional insights, the observed global disparities in BrS prevalence highlight a complex interplay of genetic, environmental, and possibly diagnostic factors. Understanding the reasons behind these geographic variations is crucial for a comprehensive global perspective on BrS.

One of the primary drivers of geographic variation is genetic predisposition. BrS is an inherited channelopathy, predominantly linked to mutations in the *SCN5A* gene, which encodes the cardiac sodium channel. While *SCN5A* mutations have been found in a significant proportion of patients with BrS, a substantial number of cases remain genetically unexplained, suggesting the involvement of other genes or complex genetic interactions (24). The prevalence and specific type of these genetic mutations vary across different ethnic groups and geographic regions. For instance, certain founder mutations may be more prevalent in specific populations due to historical migration patterns and genetic isolation (5). The higher prevalence in Southeast Asia is often attributed to a higher incidence of specific genetic variants within these populations, although the exact genetic landscape contributing to this elevated risk is still under active investigation (25).

Future studies should confirm our findings in larger, population-based cohorts. Establishing the prevalence of Brugada syndrome in our region is essential to define the disease burden and improve our understanding of its epidemiology and potential population-specific determinants. For example, detailed genetic studies in areas with high prevalence can identify common single-nucleotide polymorphisms associated with BrS. Prevalence data can also inform clinical practice by guiding targeted screening and resource allocation, while supporting the development of evidence-based national policies and healthcare planning. The yield of screening for BrS, including Proactive testing in families with sudden unexplained death and individuals with unexplained cardiac arrest, largely depends on the overall prevalence of BrS in any population.

The limitations of our study should be acknowledged. First, the single-center hospital-setting design may have introduced selection bias. We attempted to minimize this by excluding patients with presentations compatible with Brugada. In addition, many epidemiological studies have used hospital settings; thus, for comparison purposes, it remains helpful. Second, the small sample size affected precision, so a larger multicenter study is needed to confirm the results.

## Conclusions

This study suggests a high prevalence of BrS/BrP in Saudi Arabia, with a striking predominance of type 2/3 ECG patterns. However, our findings should be interpreted with caution until confirmed by larger studies. Greater awareness and education regarding BrP recognition and risk stratification are needed, in addition to further investigation into the determinants of BrS prevalence.

## Data Availability

The original contributions presented in the study are included in the article/Supplementary Material, further inquiries can be directed to the corresponding author/s.

## References

[1] Mendoza-Guazon P. Algunas notas sobre bangungut. Rev Filip Med Fam. (1917) 8:437–42.

[2] Balta A Ceasovschih A Șorodoc V Dimitriadis K Güzel S Lionte C. Broad electrocardiogram syndromes spectrum: from common emergencies to particular electrical heart disorders. J Pers Med. (2022) 12(11):1754. 10.3390/jpm12111754 36573711 PMC9697753

[3] Osher HL Wolff L. Electrocardiographic pattern simulating acute myocardial injury. Am J Med Sci. (1953) 226:541–5. 10.1097/00000441-195322650-00011 13104407

[4] Brugada P Brugada J. Right bundle branch block, persistent ST segment elevation and sudden cardiac death: a distinct clinical and electrocardiographic syndrome. A multicenter report. J Am Coll Cardiol. (1992) 20:1391–6. 10.1016/0735-1097(92)90253-J 1309182

[5] Kapplinger JD Tester DJ Alders M Benito B Berthet M Brugada J. An international compendium of mutations in the SCN5A-encoded cardiac sodium channel in patients referred for Brugada syndrome genetic testing. Heart Rhythm. (2010) 7:33–46. 10.1016/j.hrthm.2009.09.069 20129283 PMC2822446

[6] Hosseini SM Kim R Udupa S Costain G Jobling R Liston E. Reappraisal of reported genes for sudden arrhythmic death: evidence-based evaluation of gene validity for Brugada syndrome. Circulation. (2018) 138:1195–205. 10.1161/CIRCULATIONAHA.118.035070 29959160 PMC6147087

[7] Bezzina CR Barc J Mizusawa Y Remme CA Gourraud JB Simonet F. Common variants at SCN5A-SCN10A and HEY2 are associated with Brugada syndrome, a rare disease with high risk of sudden cardiac death. Nat Genet. (2013) 45:1044–9. 10.1038/ng.2712 23872634 PMC3869788

[8] Van Den Boogaard M Smemo S Burnicka-Turek O Arnolds DE Van De Werken HJ Klous P. A common genetic variant within SCN10A modulates cardiac SCN5A expression. J Clin Invest. (2014) 124(4):1844–52. 10.1172/JCI73140 24642470 PMC3973109

[9] Offerhaus JA Bezzina CR Wilde AAM. Epidemiology of inherited arrhythmias. Nat Rev Cardiol. (2020) 17(4):205–15. 10.1038/s41569-019-0266-2 31582838

[10] Vutthikraivit W Rattanawong P Putthapiban P Sukhumthammarat W Vathesatogkit P Ngarmukos T. Worldwide prevalence of Brugada syndrome: a systematic review and meta-analysis. Acta Cardiol Sin. (2018) 34(3):267–77. 10.6515/ACS.201805_34(3).20180302B Erratum in: *Acta Cardiol Sin*. (2019) **35**(2):192. 29844648 PMC5968343

[11] Antzelevitch C Yan G-X Ackerman MJ Borggrefe M Corrado D Guo J. J-wave syndromes expert consensus conference report: emerging concepts and gaps in knowledge. J Arrhythm. (2016) 32(5):315–39. 10.1016/j.joa.2016.07.002 27761155 PMC5063270

[12] Zeppenfeld K Tfelt-Hansen J De Riva M Winkel BG Behr ER Blom NA. ESC Scientific Document Group. 2022 ESC guidelines for the management of patients with ventricular arrhythmias and the prevention of sudden cardiac death. Eur Heart J. (2022) 43(40):3997–4126. 10.1093/eurheartj/ehac262 36017572

[13] Abbas H Roomi S Ullah W Ahmad A Gajanan G. Brugada pattern: a comprehensive review on the demographic and clinical spectrum. BMJ Case Rep. (2019) 12(7):e229829. 10.1136/bcr-2019-229829 31300599 PMC6626482

[14] Antzelevitch C Brugada P Borggrefe M Brugada J Brugada R Corrado D. Brugada syndrome: report of the second consensus conference: endorsed by the Heart Rhythm Society and the European Heart Rhythm Association. Circulation. (2005) 111(5):659–70. 10.1161/01.CIR.0000152479.54298.51 Erratum in: *Circulation*. (2005) **112**(4):e74. 15655131

[15] Bernardo MH Tiyyagura SR. A case of type I and II Brugada phenocopy unmasked in a patient with normal baseline electrocardiogram (ECG). Am J Case Rep. (2018) 19:21–4. 10.12659/ajcr.906464 29302023 PMC5763983

[16] Mizusawa Y Wilde AA. Brugada syndrome. Circulation. (2012) 5:606–16. 10.1161/CIRCEP.111.964577 22715240

[17] Brugada J Campuzano O Arbelo E Sarquella-Brugada G Brugada R. Present status of Brugada syndrome: JACC state-of-the-art review. J Am Coll Cardiol. (2018) 72:1046–59. 10.1016/j.jacc.2018.06.037 30139433

[18] Babaee Bigi MA Aslani A Shahrzad S. Prevalence of Brugada sign in patients presenting with palpitation in southern Iran. Europace. (2007) 9(4):252–5. 10.1093/europace/eum023 17356013

[19] Akram M AbdelWahab A Salama S Mokhtar M. Low prevalence of Brugada-type electrocardiogram in a prospective large cohort of Egyptians. Egypt Heart J. (2013) 65(4):259–63. 10.1016/j.ehj.2013.05.005

[20] Bozkurt A Yas D Seydaoglu G Acarturk E. Frequency of Brugada-type ECG pattern (Brugada sign) in southern Turkey. Int Heart J. (2006) 47(4):541–7. 10.1536/ihj.47.541 16960409

[21] Adler A Topaz G Heller K Zeltser D Ohayon T Rozovski U. Fever-induced Brugada pattern: how common is it and what does it mean? Heart Rhythm. (2013) 10(9):1375–82. 10.1016/j.hrthm.2013.07.030 23872691 PMC3832740

[22] Oe H Takagi M Tanaka A Namba M Nishibori Y Nishida Y. Prevalence and clinical course of the juveniles with Brugada-type ECG in Japanese population. Pacing Clin Electrophysiol. (2005) 28(6):549–54. 10.1111/j.1540-8159.2005.40020.x 15955188

[23] Viskin S. Brugada syndrome in children. Circulation. (2007) 115(15):1970–2. 10.1161/circulationaha.106.686758 17438161

[24] Deiman BALM Tonino PAL Kouhestani K Schrover CEM Scharnhorst V Dekker LRC. Reduced number of cardiovascular events and increased cost-effectiveness by genotype-guided antiplatelet therapy in patients undergoing percutaneous coronary interventions in The Netherlands. Neth Heart J. (2016) 24(10):589–99. 10.1007/s12471-016-0873-z 27573042 PMC5039130

[25] Brugada R Campuzano O Sarquella-Brugada G Brugada J Brugada P. Brugada syndrome. Methodist Debakey Cardiovasc J. (2021) 10(1):25–8. 10.14797/mdcj-10-1-25 PMC4051330 24932359

