# Supplementary material for: The prevalence of Brugada syndrome in patients presenting to an emergency room in Riyadh, Saudi Arabia
Source: Front Cardiovasc Med. 2026 Sep 15;13:1871087. doi: 10.3389/fcvm.2026.1871087 (PMC13635736; doi:10.3389/fcvm.2026.1871087)
Supplement: Supplementary file 1 [file Image1.pdf]

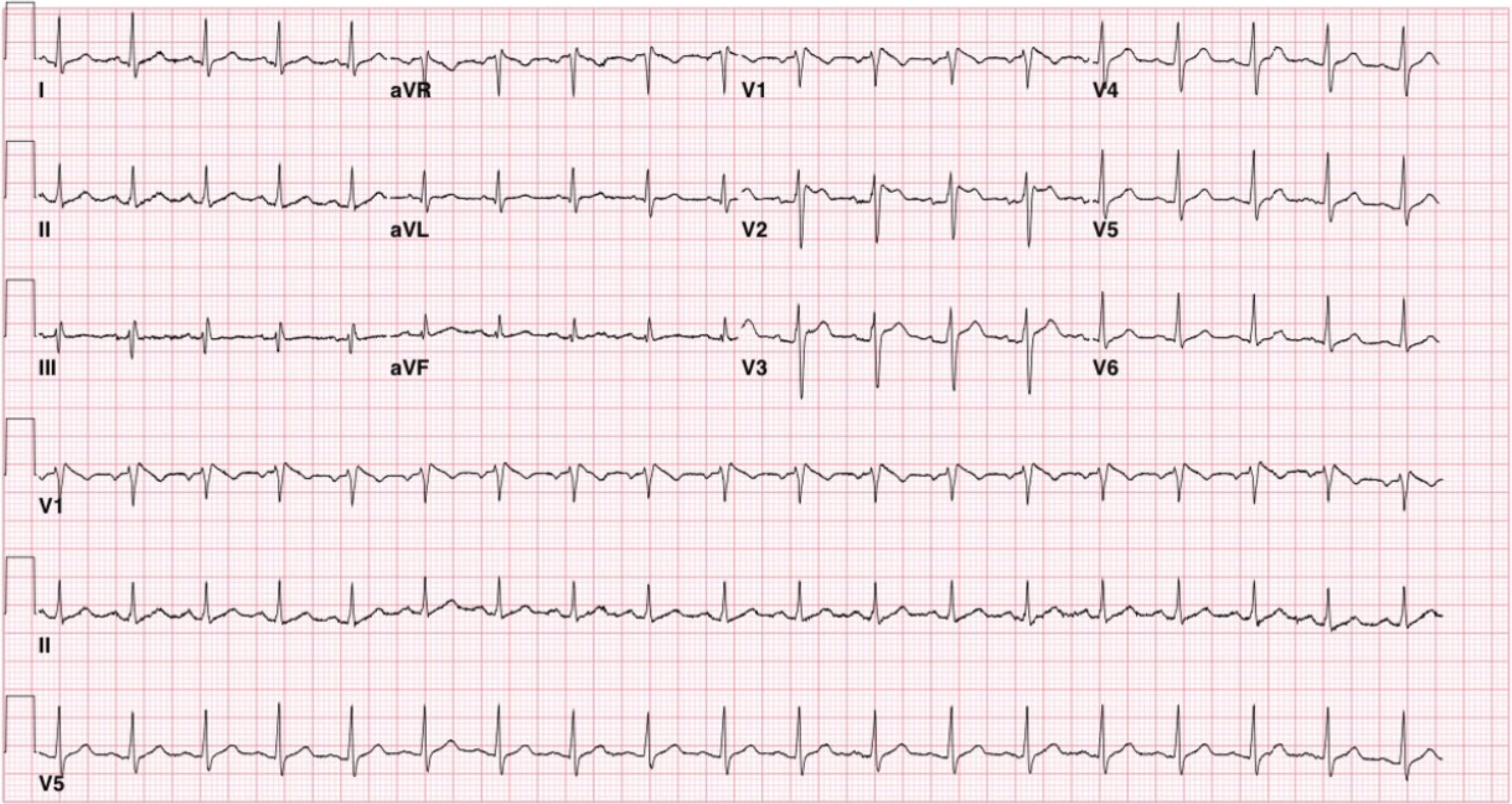

25mm/s 10mm/mV 40Hz 10.1.8 12SL 243 CID: 5 EID: 13 EDT: ORDER:
